# Agent-Based Modeling of Employee Protection-Oriented Safety Proactivity Behaviors at Small Scale Enterprises

**DOI:** 10.1155/2019/2471418

**Published:** 2019-05-19

**Authors:** Qiwei Wang, Matteo Curcuruto, Qiang Mei, Suxia Liu, Qiaomei Zhou, JingJing Zhang

**Affiliations:** ^1^School of Management, Jiangsu University, China; ^2^School of Social Sciences, Leeds Beckett University, UK; ^3^School of Management, Jiangsu University, Zhenjiang City, Jiangsu Province 212013, China

## Abstract

Although the safety production level at small scale enterprises is important for business success, critical safety interactions among the enterprises, its employees, the public, and the government have not been explained well in the literature. To address this gap, a bottom-up method of agent-based modeling is applied here that includes these key stakeholders. The study illustrates how employee protection-oriented safety proactivity behaviors, including whistleblowing and public exposure, can impact the safety production level at small scale enterprises, which are also watched by the public and regulated by the government. The results confirm that protection-oriented safety proactivity behaviors have a significant impact on the safety production levels at small enterprises through the interactions among multiple agents. The model results are validated using an employee questionnaire. The recommendation is for employees to encourage protection-oriented safety proactivity behaviors to improve safety production levels and for the public and the government to provide additional safety support.

## 1. Introduction

In China, in 2014, there were 11.6987 million small scale enterprises (SSEs), accounting for 76.57% of all enterprises and more than 70 % of all jobs [1]. In the European Union, by contrast, SSEs accounted for 98.7% of all enterprises and employed 50.2% of all employees, with large and medium enterprises accounting for only 1.3% of the total number of enterprises, although they account for 49.8% of total employment [2]. In the United States, SSEs accounted for 95% of all enterprises [3].

Obviously, SSEs are making a huge contribution to global economic development and employment. However, on-the-job fatalities and injuries continue to be problems for SSEs. Thus, it is not surprising that governments around the world are increasing their investments in safety intervention to improve safety levels. For instance, the Chinese government has implemented safety production standardization [4], and the European Union and the United States governments have implemented the Occupational Health and Safety Assessment Series (OHSAS 18001:2007) [5], the American National Standards Institute (ANSI/AIHA Z10-2012) [6], and the Occupational Safety and Health Administration (OSHA) Voluntary Protection Program (VPP) [7].

Most SSEs are unable to meet safety standards fully because of their management characteristics, such as limited resources, weak safety management practices, or a lack of safety awareness [8, 9]. SSEs have poorer occupational safety and health (OSH) conditions in general and higher accident rates than large and medium enterprises [10, 11]. Thus, their safety management characteristics may be the main obstacle to maintaining and enhancing their safety production levels [12, 13].

Many studies have stated that improving the safety climate [14–19], safety leadership [20], safety management [21], and the workplace environment [22] may reduce the rate of accidents and injuries. Moreover, employee factors, such as safety knowledge [23], safety motivation [24], and job satisfaction [25], may affect safety performance as well [23]. To improve the safety level of production, it is apparent that all the key stakeholders, the SSEs, their employees, the public, and the government must take action together.

However, although safety performance has been analyzed, the safety interactions among the SSE, its employees, the public, and the government have not been explained well in the literature. Thus, there is a need for more research that models the interactions among these key players and the influence of these interactions on safety levels in a given environment.

The rest of the paper is organized as follows.  Section 2 briefly reviews the literature. Enterprise interviews are introduced in Section 3. Agent attributes are modeled in Section 4. Simulated scenarios and model assumptions are described in Section 5. Furthermore, model results are presented in Section 6. Finally, Section 7 concludes the paper.

## 2. Literature Review

SSEs show a higher accident rate and a more dangerous workplace environment than large enterprises [26–28]. In SSEs, occupational health and safety promotions are adequately present in different methods [29–37]; however, there is a lack of comprehensive safety interventions [38–42]. The current, safety research on SSEs has focused on conceptual modeling verified with structural equation modeling methods. In order to better understand each stakeholder impacting safety production levels, the characteristics of stakeholders and their interactions must be modeled. Palaniappan et al. [43] proposed an agent-based model to explain the interaction between workers and the impacts of their safety behaviors on the safety climate and productivity. An agent-based approach was proposed by Sharpanskykh and Stroeve [44] to analyze safety culture in an air navigation service provider. Shapira et al. [45] developed an integrative model by designed weights of each risk factor in order to quantify the safety level. According to cause-effect loops, the influence of owners, designers, contractors, supervisors, and the government on safety levels was analyzed using system dynamics model [46]. The interaction among project stakeholders was simulated in a construction safety climate using agent-based modeling [47].

### 2.1. Employee Protection-Oriented Safety Proactivity Behaviors (EPOS-PB)

By definition, protection-oriented safety proactivity behaviors (EPOS-PB) are characterized by creating an observable impact for the safety of organization. The scope of this behavior is to protect the organization from negative consequences associated with safety violations and with safety standard breakdowns. Examples of EPOS-PB are stewardship [48], prevention oriented safety voice [49], and whistleblowing [50]. This study focuses on whistleblowing as EPOS-PB and its associations with public exposure and turnover phenomena.

By definition, whistleblowing occurs when employees report illegal or rule-violating behaviors to authorities outside the organization to ensure external awareness [51]. Hofmann et al. [52] defined whistleblowing from a safety perspective as reporting safety violations, instructing other colleagues to comply with safety regulations, familiarizing new team members with safety regulations, reporting colleagues who break safety regulations, and not tolerating colleagues who violate safety regulations.

Whistleblowing may encourage employers to correct wrongdoings but may also damage an enterprise's operations, reputation, and development. However, as the aim of safety whistleblowing is to prevent injuries and accidents before they happen, any enterprise operational damage may be ignored, as the results of injuries and accidents could be more destructive.

Exposure and turnover are other EPOS-PB typically associated with whistleblowing, and this study contributes to investigating how they can interact with safety proactivity. In this case, the employee exposes safety information to the public in order to protect colleagues' and his/her own health and safety; moreover, he/she chooses to escape the risky workplace.

### 2.2. Safety Production Level

Finding effective and low-cost safety systems to improve the safety production level is becoming a critical safety issue, especially in developing countries.

According to extant research [53], three factors may impact the perception of the safety level at an enterprise: employees' perceived management concerns, management's consideration of safety, and management's consideration of production. The safety level may be higher if management's consideration of safety is stronger. In contrast, the safety level may be lower if management's consideration of production is stronger. The safety level may be impacted by additional factors, including environmental factors such as sociocultural values, political decisions, economic policies, and public policies. All of these factors may affect the implementation of OSH management and the safety level [54]. Janssens et al. [53] stated that the safety level of production was measured by safety performance, the OSH situation, and the safety of the workplace environment. Isla and Díaz [55] measured the safety level based on three factors over 12 months: the level of safety in specific tasks, employees' safety compliance behavior, and operators' handling of the level of safety.

By utilizing an analytic hierarchy process (AHP), Cagno et al. [56] found that machines, operators, procedures, and the environment posed risks and caused safety issues. This risk essential method may be more practical to measure the overall safety level of production. Ayomoh and Oke [57] proposed a new method of a hybrid structural interaction matrix (HSIM) to quantify factors that may affect the safety level of production. Therefore, we propose environmental factors, such as economic policies and safety laws in our agent-based modeling (ABM).

### 2.3. Agent-Based Modeling

ABM methodology is a complex dynamic system that consists of (a) distinct autonomous heterogeneous agents with different functions; (b) behavioral rules associated with the interaction among agents, which are introduced systematically and dynamically in the system [58]. A main characteristic of ABM is that the agents update their strategy based on changing interactions and a changing environment, an action that is not possible using empirical or other mathematical methods [59, 60]. ABM has been applied in various fields in the past two decades including economics, transportation, sociology, biology, marketing, and sales among others [61]. However, studies on safety behaviors and safety production levels are typically empirical or linear, limiting comprehensive analysis. ABM applies a bottom-up method that defines different agent strategies and attributes and builds properties of the environment, allowing analysis of the interactions among agents in different periods [62].

In addition, ABM can simulate what-if scenarios, allowing evaluations of the different options to shape enterprises strategies [58]. For instance, ABM can be used to propose a model and then change parameters to determine the responses to the changes. Awwad et al. [47] simulated interactions among project stakeholders within a construction safety climate during both the bidding and construction phases. Lu et al. [63] analyzed the interactions among a worksite, construction employees, and various types of safety investments to identify the interplay between safety investment and safety performance. In this study, we utilize ABM to analyze how employee EPOS-PB may have different impacts on the safety production level under specific environments.

### 2.4. Safety Management Characteristics of SSEs

Relevant studies on SSEs have shifted from addressing safety hazards to safety intervention that may reduce accident rates [9]. Although SSEs are heterogeneous, they share common business characteristics concerning the delivery of products and services, according to certain productivity standards and priorities [64]. Based on studies of SSEs, SSEs employees may face a more risky workplace environment [65, 66], lower guarantee of OSH, and lower effective implementation of safety regulations and laws compared with large enterprises [67, 68].

Owners of SSEs often play the role of managing safety; thus, all safety issues are personal decisions rather than based on specific directives [69]. As owner-managers of SSEs often take total responsibility for both production and safety, they have little time to solve safety issues [70]. Obviously, the safety attitude of the owner-manager has a significant impact on the safety level of production. At the same time, owner-managers must deal with government safety regulations and laws that may create a negative effect on OSH and safety management [67].

As these businesses are heterogeneous, the owner-managers of the SSEs will have different attitudes and strategies for safety regulations and inspections that affect the safety levels [71]. Safety information is often limited and owner-managers may lack the necessary experience or responses to solve safety issues and face inspections [72]. Because of insufficient safety knowledge, resources, and funds, there may be more safety issues among SSEs than in large and medium enterprises [65, 72]. Recent studies in literature suggest that managerial intervention is aimed at improving safety information sharing; a better knowledge of safety regulation guidelines and employees' safety participation in the management of safety can help SSEs to enhance the quality of safety standards and the maturity of the safety system [48, 73]. According with this recent trend in literature, Mei et al. [74] also suggest that stimulating safety proactivity behaviors could positively impact safety management of SSEs.

Overall, owner-managers of SSEs often have a poor understanding of OSH regulations and legislation, have limited capacity to identify risks and hazards, and may have negative attitudes to safety inspections [75]. In this context, it is necessary to equate safety and production.

Obviously, the improvement in safety levels at SSEs relies not only on the efforts of employees but also on support from the public and the government. In this study, we utilize ABM to analyze the interactions among employees, SSEs, the public, and the government.

## 3. Enterprise Interviews

This study gathered data on the safety production levels in Chinese SSEs through semistructured interviews with 105 high-risk SSEs. The literature review and reality confirmed that there were more accidents and injuries among SSEs than among large and medium enterprises. According to safety regulations, owner-managers of SSEs are obliged to report safety occurrences and ensure safety reform. If accidents and injuries happen due to illegal production activities, they may also receive a punishment such as a fine or closure. Particularly, reports on safety occurrences are useful for safety analysis (e.g., statistics of accident or injury ratios and safety improvement for SSEs). Although safety reports are obligatory, in reality, not all occurrences are reported in a timely manner by owner-managers. At the same time, due to a lack of safety investment, SSEs suffer from a low safety level of production. As their emphasis is on survival and development, most SSEs fail to achieve the required safety standards. Therefore, in this study, in the context of SSEs, we model how they can make proper safety investment decisions and increase their safety level.

The survey results indicated that most SSEs showed strong performance on the safety production level, which meant that their frequency of safety occurrences was significantly low. At the same time, safety investments among the SSEs were insufficient, not only due to a lack of safety awareness but also because of realistic constraints. Owner-mangers generally chose to conceal safety occurrences rather than report them because if they reported them, they would be required to compensate employees and pay a penalty to the government. Thus, insufficient safety investment could cause an adverse chain reaction. To reduce safety investment, a small number of SSEs even chose unsafe production. Employees facing safety risks have the civic right to expose or whistle blow, although most of them chose to keep silent. For these reasons discussed above, employee EPOS-PB can play a significant role in improving safety production levels. In order to understand how to stimulate and support them, in the next section, we propose how to design and develop a formal model based on ABM methodology. In doing this, we will take into consideration all the major attributes of the agents contextually involved: SSE; employees; public; government.

## 4. Modeling Agent Attributes

### 4.1. Employee Attributes

The safety production levels at SSEs affect the employees' OSH conditions. According to extant research [57], when the safety level of production is low, the employee-perceived OSH level will also be relatively low. Thus, employee OSH will be threatened by risks and hazards that affect their workplace environment. In contrast, when the safety level is high, the employee-perceived OSH level will be relatively high; the OSH level of employees should be maintained within a stable range. Therefore, the employee-perceived OSH level related to safety level of production of SSEs *i* in *t* period is as follows:

(1) $$\begin{array}{c} OSH\;\;level=\theta *Safety-level\;\;of\;\;production_{i,t} \end{array}$$

where *θ* is the random coefficient of the employee-perceived OSH level in the range [0.8, 1.2]. Based on cognitive bias, some employees may have a low perception of the safety level even if the safety level is high, while some employees may have a high perception of safety level even if the safety level is low; some employees may have the same perception as the actual safety level. Thus, the model sets a minimum value of 0.8 to represent a low-bias perception, a maximum value of 1.2 to represent a high-bias perception, and the value of 1 to represent the same perception.

The value of employee-perceived OSH level can be used to determine employees' real value of safety production efficiency. This value means that employees perform daily production activities under a fixed level of safety, and the degree of the safety level of production will decide the safety efficiency for the employees.

(2) Safety  production  efficiency=M∗OSH  level∗η+δ

where *M* = 0.22, *η* = 0.4, and *δ* = 0.6. Small coal mine enterprises were chosen to determine the value of *M*, which represents the efficiency coefficient without the influence of the OSH level. *η* and *δ* were determined by the system design and repeated simulation experiments.

### 4.2. Public Attributes

Social Networking Services (SNS) and Mass Media and Politics (MMP) offer opportunities for employees to expose safety information to the public. Thus, the public can obtain information directly from employees instead of owner-managers or safety news reports. An enterprise's public reputation value can impact the purchase intention of customers and, subsequently, overall sales. Therefore, the sales of SSEs can be calculated as follows:

(3) Salesi,t=Salesi,t−1SCt≥R1Salesi,t−1∗SCt∗0.5+0.6R0≤SCt<R1Salesi,t−1∗SCt∗0.167+0.80<SCt<R0

where *SC*_*t*_ is the public reputation value in *t* period, and the threshold values of *R*_0_ and *R*_1_ represent the degree of reputation value from employees, where *R*_0_ = 0.6 and *R*_1_ = 0.8. When the reputation value is low, sales will be negatively influenced, and when the reputation value increases, sales will increase correspondingly. When the public reputation is more positive, sales will meet normal market demand. The threshold and other values in (3) were determined based on the system design and repeated simulation.

### 4.3. Government Attributes

Employees have civic rights to blow the whistle on SSEs that break safety laws and regulations or disobey OSH terms. Whistleblowing safety behavior can attract government safety attention; the national administration of production supervision has the responsibility to regulate safety production standardization and implement OSH policy. Meanwhile, the government evaluates the safety level of production at SSEs based on political and systematic standardization, employee reports, and public response. Consequently, the government puts in place a relevant political strategy according to different levels of safety. Ultimately, when the level of safety is below the standard, the government will require SSEs to identify risks and hazards and improve the workplace environment or be penalized if no action is taken. Similarly, when the level of safety meets or exceeds the requirement, the government will reward SSEs to maintain their performance. The reward and penalty system is shown as follows:

(4) $$\begin{array}{c} System_{i,t}=\left\{\begin{array}{cc} R, & System_{i,t}<T_{0}\\ 0, & T_{0}\leq System_{i,t}<T_{1}\\ P, & T_{1}\leq System_{i,t}<T_{2}\\ B, & T_{2}\leq System_{i,t} \end{array}\right. \end{array}$$

where the threshold values of *T*_0_, *T*_1_, and *T*_2_ represent the reward and penalty standards based on the level of safety; *T*_0_ = 0.6, *T*_1_ = 0.7, and *T*_2_ = 0.8; *P* and *B* are the reward values and *R* is the penalty value. According to safety laws and survey results, the model determined *B* = 50000, *P* = 5000, and *R* = 10000 and the threshold values of *T*_0_, *T*_1_, and *T*_2_. It is difficult to examine the effects of different levels of rewards and penalties because of the complex system design.

Furthermore, the government has the function of tax regulation. In order to encourage SSEs to improve the level of safety, the government adjusts the tax rate according to the level of safety as follows:

(5) $$\begin{array}{c} Tax\;\;rate_{i,t}=\left\{\begin{array}{cc} 0.2, & System_{i,t}<T_{0}\\ 0.15, & T_{0}\leq System_{i,t}<T_{1}\\ 0.1, & T_{1}\leq System_{i,t}<T_{2}\\ 0.05, & T_{2}\leq System_{i,t} \end{array}\right. \end{array}$$

where *System*_*i*,*t*_ represents the evaluation result of the safety level of production. Because of the complex system design, the tax rate was determined by the system, although it was difficult to show the tax rate in different simulations.

Finally, based on the safety policy, the government evaluates the reliability of the whistleblowing information and once confirmed, it rewards the employee. When *GC*_*i*,*t*_ < *T*_0_, the whistleblowing reward value is shown as follows:

(6) Whistleblowing  reward  value=Safety  production  efficiency+0.1

Equation (6) is utilized in the system, so it cannot be found in other equations in the model.

### 4.4. SSE Attributes

SSEs have functions such as production, selling, and profit. During the process of production, the safety level of production will affect the OSH conditions of employees and then affect employee production efficiency. However, because of the characteristics of SSEs, most SSEs have a low level of safety compared to large and medium enterprises; thus, the safety level will significantly influence production. The calculation method is from the safety investment model described by Lu et al. [63].

(7) $$\begin{array}{c} Safety-level\;\;of\;\;production_{i,t}=\frac{\beta K_{i,t}}{\left(1+\beta K_{i,t}\right)} \end{array}$$

where *K*_*i*,*t*_ is the safety investment and *β* is the control coefficient, and *β* = 0.33 according to the system design and repeated simulation experiments.

Safety and productivity are two key factors for SSEs. Most SSEs may consider safety and productivity as conflicting issues [76]. Some SSEs may choose to reduce safety investment and produce in an unsafe way as they consider profits more important than safety [77]. According to the model, the safety production function is decided by the fixed product price *FP*_*i*,*t*_, working time *T*, employee safety production efficiency *SV*, the number of employees *EN*, and sales *PS* [78] as follows:

(8) $$\begin{array}{c} Safety\;\;production_{i,t}=\overset{n}{\underset{i=1}{\sum }}FP_{i,t}*T*SV*EN*PS \end{array}$$

where *FP*_*i*,*t*_ is 300 per unit, *T* is 3 months, *EN* is 40, and *WE* is 3000 CNY per person.

### 4.5. Self-Learning Algorithm of SSEs

Based on the economic situation, the model includes a self-learning SSE mechanism and the characteristic of bound rationality. To represent the subjectivity of SSEs, this study applies the self-learning algorithm so that the SSEs will change their safety investments according to the periodic evolutionary trend of the safety level of production. This self-learning algorithm includes intellectuality and automaticity, which maintain the status of effectiveness. Thus, it mimics more closely a realistic situation and enables SSEs to make decisions to change their level of safety investment.

SSEs will make decisions after the end of each period based on two results: first, by evaluating the previous period, they decide if the safety level of production is increasing, decreasing, or unchanged during the current period, compared to the previous one; and second, they decide if profits are increasing, decreasing, or unchanged during the current period, compared to the previous one. After the two decisions, SSEs will adjust the probability of their safety investment (see Table 1).

The system defines the probability vector of the safety investment adjustment as *p* = (*p*^*d*^, *p*^*i*^, *p*^*c*^), where *p*^*d*^ represents the probability of a decreasing safety investment in the next period, *p*^*i*^ represents the probability of an increasing safety investment in the next period, and *p*^*c*^ represents the probability of an unchanged safety investment in the next period, where *p*^*d*^ + *p*^*i*^ + *p*^*c*^ = 1.

A change in the current strategy is based on the previous change in the safety level, and the change process reflects the self-learning mechanism. When the current strategy is completed, the system will randomly generate a number *R* between 0 from 1 to decide which strategy the SSE applies.

(9) $$\begin{array}{c} K_{i,t}=\left\{\begin{array}{cc} K_{i,t-1}-\Delta I_{1}, & 0\leq R<p^{d}\\ K_{i,t-1}-\Delta I_{2}, & p^{d}\leq R<p^{d}+p^{i}\\ K_{i,t-1}, & p^{d}+p^{i}\leq R\leq 1 \end{array}\right. \end{array}$$

where Δ*I*_1_ and Δ*I*_2_ represent the increment and decrement of the safety investment. These two numbers are related to the safety level of production. Δ*I*_2_ of high and medium safety level SSEs is greater than the safety investment of low safety level SSEs, and Δ*I*_1_ of low safety level SSEs is greater than the safety investment of high safety level SSEs.

The safety production cost *C*_*i*,*t*_ can be shown as the relationship of the labor cost *LC*_*i*,*t*_ and the production cost *PC*_*i*,*t*_, and the safety investment *K*_*i*,*t*_.

(10) $$\begin{array}{c} C_{i,t}=\overset{n}{\underset{i=1}{\sum }}LC_{i,t}+K_{i,t}+PC_{i,t} \end{array}$$

Finally, based on the above analysis, the profit of the SSEs can be shown as follows:

(11) Fi,t=∑i=1n1−Tax  ratei,tSafety  productioni,t−Ci,t+Systemi,t

### 4.6. Modeling Agent Interactions

As stated previously, whistleblowing can take different forms such as exposing safety information to the public and reporting the owner-managers who do not comply with the standards of safety regulations. When the public learns about safety incidences, the reputation of SSE is affected, which thereby influences the consumer purchase intention and, thus, the sales of products. When the government receives a safety report, it implements a reward and penalty system according to the level of safety and also rewards whistleblowing behavior. In actual practice, employees could choose to leave or ask for a raise if they face poor safety conditions and owner-managers are not willing to address them.

**Figure 1:**
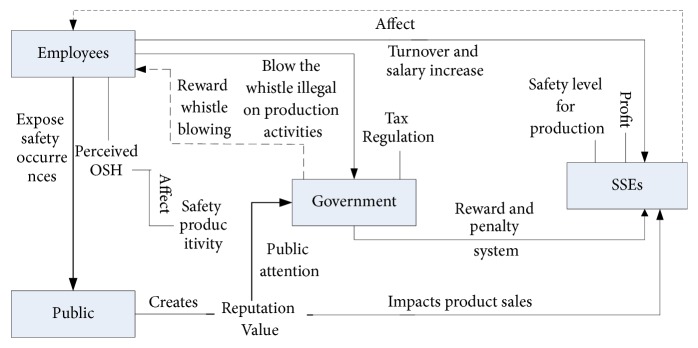
Interaction process among agents.

ABM includes three components: (1) properties, behaviors, and the environment of agents, (2) each agent's interactions with the environment, and (3) the interactions among different agents [79]. The schematic of the interaction process among these agents is shown in Figure 1.

## 5. Simulated Scenarios and Model Assumptions

### 5.1. Simulated Scenarios

The environment is defined based on the characteristics of the SSEs and the parameters are as close to reality as possible including the number of employees, enterprise scale, the safety level of production, safety production efficiency, and gross safe production.

Based on the fluctuation of the safety level of production, this paper presents five scenarios. The five scenarios simulate the interactions among the agents, including employee EPOS-PB, the fluctuation of the reputation value of the public reputation value, and the dynamic regulation of the government. The scenarios aim to simulate reality to arrive at the optimal strategy.

The five scenarios design the interaction between the employees and the SSEs. The safety level of production affects the OSH level of employees. The safety production efficiency of employees is dependent on the perceived OSH level and employees choose different strategies about production activities. In Scenario 1, employees take no action to affect the safety level of production and do not voice their safety concerns in order to keep their positions. In Scenario 2, based on the perceived OSH level, employees expose safety occurrences through SNS and MMP, rather than blow the whistle to a government entity. In Scenario 3, employees directly blow the whistle to the government without relying on SNS and MMP. In Scenario 4, employees choose to expose safety occurrences to the public and blow the whistle; thus, the public and the government have a dual-effect in regulating the safety level of production. Finally, Scenario 5 reflects a more realistic situation; specifically, employees choose to leave, or if they insist on staying, they demand a raise, as the safety regulation of both the public and the government shows a non-immediate effect and owner-managers refuse to improve the workplace environment and OSH level for employees. In addition, high safety level SSEs will be able to recruit workers more easily; a current issue for SSEs is difficulty in recruitment. Thus, low safety level SSEs will find it difficult to recruit employees.

### 5.2. Model Assumptions

Based on the literature, SSEs must solve OSH issues and safety investment will impact the safety level of production, which will then impact production and sales. Focusing on production, the agents have different attributes, for instance, the government can both inspect and regulate safety. When the level of safety reaches high or medium, the SSEs receive a reward; on the other hand, if there is a low level of safety, SSEs will be penalized, required to fix the problem, or shut down [80]. With the rapid development of social media, employees could expose the level of safety information to the public through SNS and MMP. In this way, the public will be made aware of the safety level of production of the SSEs and could affect the safety attitudes of owner-managers. ABM can be used to analyze the evolutional rules of the safety level under different environments with different interactions among the agents. The model assumptions are as follows:

1. To identify the interactions among the SSEs and other agents, we assume that the number of SSEs is fixed and the number of employees is based on the characteristics of the SSE.
2. To simplify the multi-dimensional safety level of production, we consider the degree of safety investment as a key factor that affects the safety level of production [63].
3. In the system, SSEs sales are impacted by the public and product prices are based on the SSE scenario.
4. Based on the characteristics of the SSEs and the design of the system, we assume that the increase and decrease in the ratio of the safety investment are controlled in a reasonable range.
5. According to China safety production standardization [81], we assume that SSEs are divided into four types: first-degree safety level of production *c*_1_, second-degree safety level of production *c*_2_, third-degree safety level of production *c*_3_, and fourth-degree safety level of production *c*_4_. *c*_1_ and *c*_2_ represent a high and medium level of safety while, *c*_3_ and *c*_4_ represent meeting the standard and failing to meet the standard, respectively.

## 6. Model Results

To highlight the purpose of the experiment and the comparability of agents, the SSE safety level of production was divided into four sublevels. In the model, the maximum and minimum safety production standardization values were *SD*_max_ and *SD*_min_, respectively, with *SD*_max_ = 100 and *SD*_min_ = 0. The government evaluation values of safety production standardization were defined as *S*_max_ and *S*_min_. Thus, the safety level of production of SSEs was *s* ∈ (*s*_min_ = *S*_min_/*SD*_min_, *s*_max_ = *S*_max_/*SD*_max_).

(12) $$\begin{array}{c} c=\left\{\begin{array}{cc} 1, & \frac{8}{10}\left(s_{max}+s_{min}\right)\leq s<s_{max}+s_{min}\\ 2, & \frac{7}{10}\left(s_{max}+s_{min}\right)\leq s<\frac{8}{10}\left(s_{max}+s_{min}\right)\\ 3, & \frac{6}{10}\left(s_{max}+s_{min}\right)\leq s<\frac{7}{10}\left(s_{max}+s_{min}\right)\\ 4, & s_{min}<s<\frac{6}{10}\left(s_{max}+s_{min}\right) \end{array}\right. \end{array}$$

Two experiments were conducted to simulate the interaction among agents. The different scenarios illustrate the evolutionary trend of SSEs and the profit of the SSEs based on the interactions.

### 6.1. Evolutionary Rules of the Safety Level of Production in Different Scenarios

**Figure 2:**
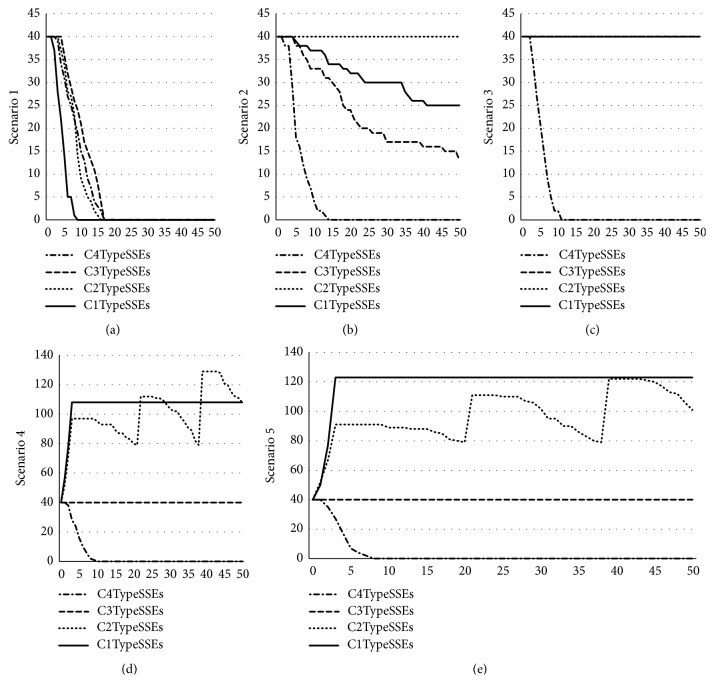
Evolutionary number of SSEs in different scenarios.

The internal interactions between employees and SSEs and the external interactions with the public and the government show diversity and complexity. The safety level of production will present different forms. Thus, first, we simulated the evaluation number of the SSEs to identify the optimal strategic scenario. Figures 2(a), 2(b), 2(c), 2(d), and 2(e) show the simulation trends of scenarios 1, 2, 3, 4, and 5, respectively.

Based on the simulations, in Scenario 1, where employees take no action, the evolutionary level of all types of SSEs was the lowest compared with the other four scenarios. When reaching a specific period, *c*_1_ high-level safety SSEs reached zero quickly. Second, in Scenario 2, where employees expose information to the public, the evolutionary trend of *c*_4_ low-level safety SSEs was obviously faster than that in Scenario 1. The *c*_2_ medium-level safety SSEs maintained a steady trend and the highest position. The *c*_1_ and *c*_3_ (high- and standard-level) SSEs showed a similar evolutionary trend, which decreased with the periods. Third, in Scenario 3, where employees blow the whistle, the decreasing rate of the *c*_4_ low-level safety SSEs was similar to Scenario 2. However, *c*_1_, *c*_2_, and *c*_3_ (high-, medium-, and standard-level) SSEs showed a similar decreasing rate after the periods. Finally, Scenario 4, where employees expose and blow the whistle, and Scenario 5, where employee choose to leave or ask for a raise, show similar evolutionary trends. Specifically, the *c*_4_ low-level safety SSEs show a closely related rate of decrease in both scenarios. Due to the addition of the agents, the interactions become positive; therefore, the fluctuation of *c*_2_ medium-level safety SSEs was greater in both scenarios. After a short period, the trend of *c*_1_ high-level safety SSEs becomes relatively stable and remains higher than the others. However, the SSEs are more likely to evolve into the *c*_1_ type.

### 6.2. Rules of Profit Fluctuation of SSEs in Different Scenarios

To explain the profit trends, we constructed different scenarios to show how SSEs develop through the interactions of the agents. The SSE profit will change based on different agent actions; each scenario simulates one situation. As the interactions have five different agents, we cannot use a unified indicator to evaluate the changing profits, but we can identify the changing trend by comparing the interactions among the agents.

**Figure 3:**
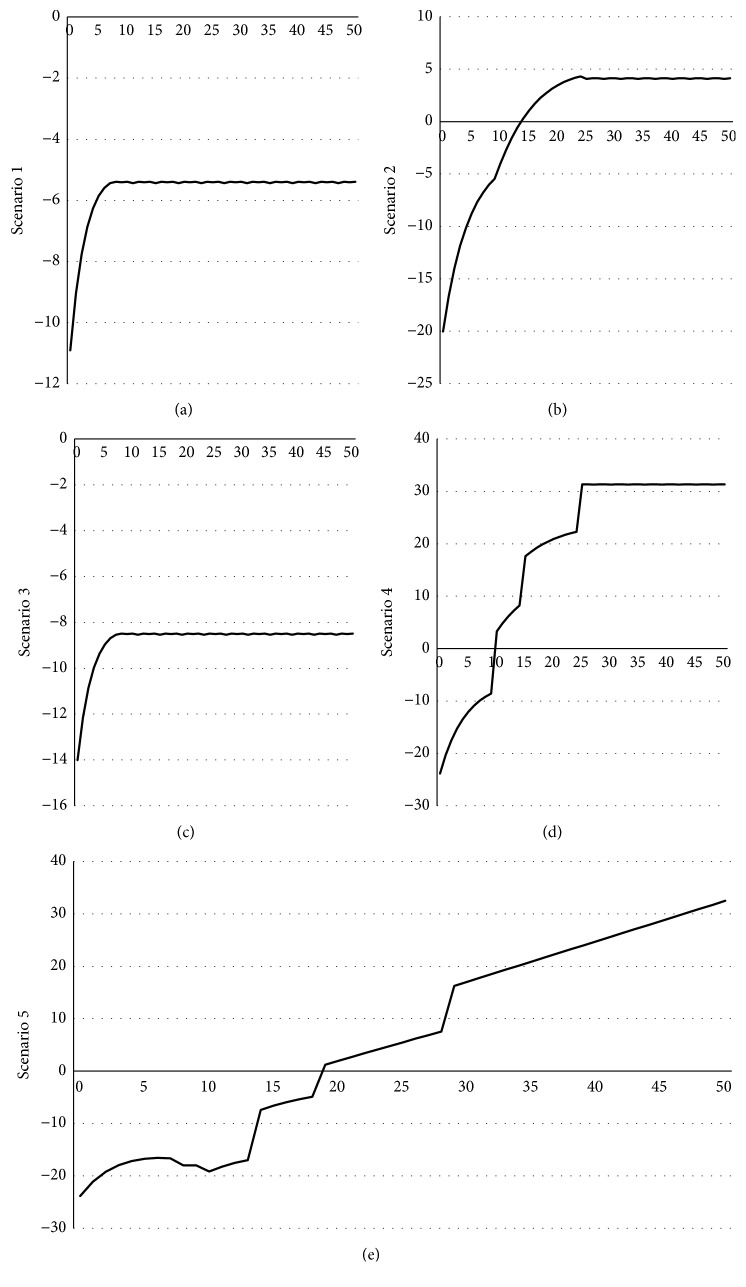
Fluctuating profit of SSEs in different scenarios.

First, the SSE profit was the lowest in Scenarios 1 and 3, (no action and whistle blowing, respectively) relatively, among all scenarios. SSE with low safety level went bankrupt and exited the market. During the period, profit reached a high point and then began to decline. However, the profit was still far below zero. Second, the increase in the profit trend was faster in Scenario 2 (public exposure) when the profit peaked and then started to decrease. However, the profit in Scenario 2 was smaller than in Scenarios 4 and 5. Finally, Scenario 4 (exposure and whistle blowing) showed a fast increasing rate of profit initially, which then became more stable over the periods. In contrast, in Scenario 5 (employees leave or ask for a raise), the profit showed some fluctuation initially, then a rapid increase. In the middle and final periods, the profit trend continued to rise steadily. Figures 3(a), 3(b), 3(c), 3(d), and 3(e) show the simulation trends of scenarios 1, 2, 3, 4, and 5, respectively.

The simulation results show that when SSEs only consider productivity, employees remain silent about working conditions and the public and the government neglect supervision and regulation, with the result that SSEs have a lower level of safety. Under these conditions, there is no incentive to improve the safety level of production; thus, SSEs will remain at their current safety level of production and there is less probability that the level can evolve to a high or medium level. Furthermore, at a lower level of safety, employees suffer more risks and hazards. When injuries, accidents, or fatalities occur, the employees, reputations, and sales of the SSEs are affected due to public response and potential administrative penalties from the government. Thus, in the simulation, the SSE profit was extremely low when SSEs only focused on productivity and ignored the safety production. When the model added the reward and penalty system, we observed the beginning of the intention to improve safety production levels among the SSEs. When employee EPOS-PB were included in the model, the safety level of production reached its highest point and remained steady over the long term.

### 6.3. Model Validation

The level of validity was determined by the results of the surveys and of the safety level of production of the SSEs. The questionnaire comprised a set of statements about employee EPOS-PB and safety level of production at the SSEs (Table 2). The questionnaire results showed the level of perception of employees on a scale from 1 (strongly disagree) to 5 (strongly agree). Employee beliefs on the impact of protective-oriented safety proactivity on the safety level of production and related options were determined from the simulation scenarios stemming from the safety level of production survey results.

According to the comparison between the simulation results and the survey results, we introduced five classes of safety values: Very low, Low, Medium, High, and Very high. According to the values of the simulated scenarios, the model and survey results are shown in Table 3.

The comparison between the model and survey shows that the results are consistent. However, Scenario 5 could not be verified by the survey because we could not obtain information from employees who had already left their jobs.

To validate the agent-based model between the agent and model scenarios, the results of the survey were used. The purpose of the survey was to acquire the employee perceptions through interviews with actual SSE employees. The model was validated through the comparison of the model and the survey.

Simulated scenario results were matched with the survey results. Specifically, Scenario 1 shows the lowest impact on the safety level of production. Scenario 2 shows a medium impact due to the interactions between employees and the public. Scenario 3 shows a low impact as SSEs suffered a penalty or political punishment. Scenario 4 shows a high impact with effects from both the public and the government. Scenario 5 shows the highest impact not only on the basis of Scenario 4, but also due to the more EPOS-PB.

## 7. Conclusion

Currently, few researches focus on proactivity safety based on the method of ABM. Goh and Ali [82] proposed a hybrid simulation framework of discrete event simulation, system dynamics, and agent-based simulation to demonstrate the relation between safety behavior and construction safety management. Lu et al. [63] used agent-based model to analyze safety performance on a construction site based on a complex system defined by interactions among a worksite, workers, and safety investment. Agent-based approach was utilized to analyze the relation between an air navigation service provider and organizational safety culture by Sharpanskykh and Stroeve [44].

Although the existing models are adequate in modeling stakeholders that affect safety levels, they fall short in modeling the integrative agents whose behaviors and attributes impact the safety production level of SSEs. This objective of the study is to utilize a bottom-up method of agent-based modeling (ABM) to study EPOS-PB by combining the stakeholders in the system and simulating their interactions. ABM is an effective technique to develop computational models of SMEs safety of production and dynamic interactions. Other methodological approaches cannot show the advantages because most of them are linear and non-dynamic. The simulation results show a dynamic evolution of the safety level. They also point to low-cost and highly effective safety interventions for SSEs and optimal safety strategies for policy makers.

The model validation was performed based the comparison between the simulation and survey results. The comparison showed that most model results were consistent with the results of the employees' survey workshop. The survey results were used not only for the model input but also for the validity of the model results. However, Scenario 5 cannot be validated because of reality constraints.

Based on the simulations of the interactions among the different agents, one significant finding was that rather than remaining silent, if employees pursue EPOS-PB, they can help improve the safety level of production at their SSE. Another significant finding was that SSEs should not only target productivity but also a high safety level of production. As profit is the key goal necessary to survive and develop, owner-managers should equally value both safety and production, instead of having to reduce safety investment to maintain profits.

At a practical level, the findings suggest that safety interventions should aim at focusing on EPOS-PB and the responsibility of the public and the government, which becomes the most effective in improving safety level production of SSEs. Specially, employees should consider safety as the core and basic requirement; they should not only blow the whistle immediately on illegal and unsafe production activities to the government but also report safety information to the public. When facing owner-managers' refusal in improving safety levels, employees should leave the job or demand for a raise to make additional efforts to improve the workplace environment. The public currently has few channels for employees to expose safety occurrences. Therefore, the public should offer specific SNS and MMP to allow employees to report safety issues. Furthermore, regarding policy makers, employees may experience ethics pressure if they choose to be a whistleblower. Thus, the government should install anonymous telephone hotlines and conceal whistleblowers' information. The national government should simultaneously encourage employees to blow the whistle and formulate a series of laws and policies to protect whistleblowers. In addition, a more humanized reward and penalty system can be designed; for instance, implementing a purely monetary awards and punishment mechanism could provide safety assistance to SSEs, such as purchasing safety services in a discount, pressuring owner-managers to implement OSH policy in a gentle way. The government should not only reward whistleblowers, but also provide policy guarantee to employees to make them feel safe about their workplace environment and OSH condition. Finally, the standard evaluation of the safety level for SSEs should be less strict, compared with that for large and medium enterprises.

Improving the safety levels in SSEs is not only dependent on the efforts of owner-managers but also on the combined efforts of employees, the public, and the government. The results from these simulations can be used to provide the public, policy makers, and owner-managers with information on how employee EPOS-PB can affect safety production levels for SSEs. The public, policy makers, and university research teams can practically use this ABM model. Specifically, the results can give the changing safety trend of SSEs for the public, different ratio regulations and safety regulation for policy makers, and safety researches for university research teams.

The study has some limitations. First, the ABM is abstracted from real-world SSEs and it cannot simulate fully all factors related to the current market situation. Second, the model could be better integrated. Finally, this study does not consider additional agents, such as labor unions or financing institutions.

Therefore, in future work, the ABM model could be modified to add more agents and build more impact factors to make the ABM model more realistic.

## Figures and Tables

**Table 1:** Adjustment probability of safety level of production.

Safety investment	Profit	Probability
Increase	Increase	(*p*^*d*^ − *ε*/2, *p*^*i*^ + *ε*, *p*^*c*^ − *ε*/2)
	Decrease(or no change)	(*p*^*d*^ + *ε*/2, *p*^*i*^ − *ε*, *p*^*c*^ + *ε*/2)
Decrease	Increase	(*p*^*d*^ + *ε*, *p*^*i*^ − *ε*/2, *p*^*c*^ − *ε*/2)
	Decrease (or no change)	(*p*^*d*^ − *ε*, *p*^*i*^ + *ε*/2, *p*^*c*^ + *ε*/2)
Unchanging	Increase	(*p*^*d*^ − *ε*/2, *p*^*i*^ − *ω*/2, *p*^*c*^ + *ε*)
	Decrease (or no change)	(*p*^*d*^ + *ε*/2, *p*^*i*^ + *ε*/2, *p*^*c*^ − *ε*)

**Table 2:** The questionnaire about employee EPOS-PB and safety level of production at the SSEs.

Item	Description
Q1	I believe that taking no action will positively impact the safety production level
Q2	I believe that exposing safety occurrences will positively impact the safety production level
Q3	I believe that blowing the whistle on illegal production behavior will positively impact the safety production level
Q4	I believe that both exposing and blowing the whistle will positively impact the safety production level

**Table 3:** The values of the simulated scenarios and the survey questionnaire.

Simulated Scenarios	Model	Survey	Mean	SD
Scenario 1: Average impact of employees taking no action	Very low	Very low	1.78	0.797
Scenario 2: Average impact of employees exposing safety occurrences	Medium	Medium	2.48	0.588
Scenario 3: Average impact of employees blowing the whistle on illegal production behavior	Low	Low	3.63	0.615
Scenario 4: Average impact of employees both exposing and blowing the whistle	High	High	4.04	0.767
Scenario 5: Average impact of employees adding turnover and demanding for a raise on the basis of Scenario 4.	Very high	-		

## Data Availability

The data used to support the findings of this study are available from the corresponding author upon request.
